# Supplementary material for: Radiative cooling assisted self-sustaining and highly efficient moisture energy harvesting
Source: Nat Commun. 2024 Jul 19;15:6100. doi: 10.1038/s41467-024-50396-9 (PMC11271565; doi:10.1038/s41467-024-50396-9)
Supplement: Supplementary file 1 — Supplementary Information [file 41467_2024_50396_MOESM1_ESM.pdf]

Supplementary Information

Radiative cooling assisted self-sustaining and highly efficient moisture energy harvesting

Chenyue Guo[1], Huajie Tang[1], Pengfei Wang[2], Qihao Xu[1], Haodan Pan[1], Xinyu Zhao[1], Fan Fan[1], Tingxian Li[2]* & Dongliang Zhao[1, 3, 4]*

[1]School of Energy and Environment, Southeast University, 210096 Nanjing, China.

[2]Institute of Refrigeration and Cryogenics, School of Mechanical Engineering, Shanghai Jiao Tong University, 200240 Shanghai, China.

[3]Institute of Science and Technology for Carbon Neutrality, Southeast University, 210096 Nanjing, China.

[4]Institute for Carbon Neutral Development, Southeast University, 210096 Nanjing, China.

These authors contributed equally: Chenyue Guo, Huajie Tang, Pengfei Wang.

*Corresponding author. e-mail: dongliang_zhao@seu.edu.cn; litx@sjtu.edu.cn

This file includes:

Supplementary Figures 1–25

Supplementary Notes 1–9

Supplementary Table 1

Supplementary References

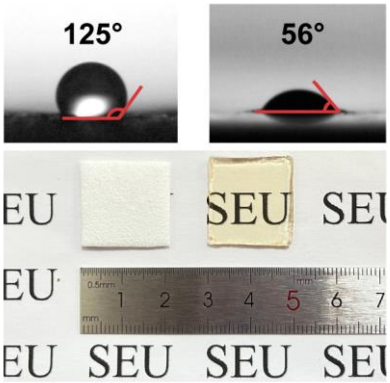

Supplementary Fig. 1 Photograph and water contact angles of the porous P(VdF-HFP) layer (left) and the ionic hydrogel layer (right).

Supplementary Note 1

As shown in Supplementary Fig. 2, the O 1*s* peak can be divided into three electronic states at 531.8, 532.5, and 533.2 eV, corresponding to P═O, P–O, and C–O[1]. The P 2*p* peak can be fitted with two peaks at 133.5 eV (P═O···H–O–P) and 134.3 eV (P═O···H–O–C), revealing the existence of intermolecular and intramolecular hydrogen bonds between PA and PVA[2].

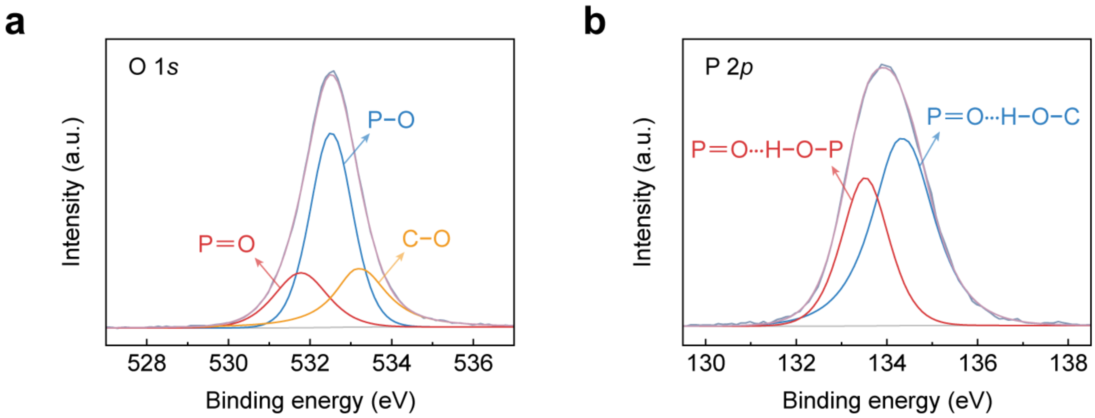

Supplementary Fig. 2 X-ray photoelectron spectroscopy (XPS) of the ionic hydrogel and the corresponding high-resolution spectroscopy of **a** O 1*s*, **b** P 2*p*.

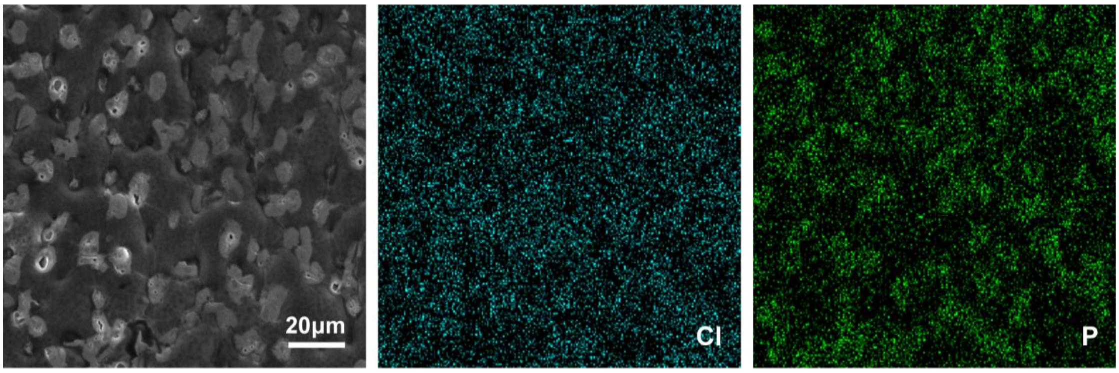

Supplementary Fig. 3 The cross-sectional SEM image of the dehydrated ionic hydrogel as well as the corresponding EDS mapping of Cl and P elements.

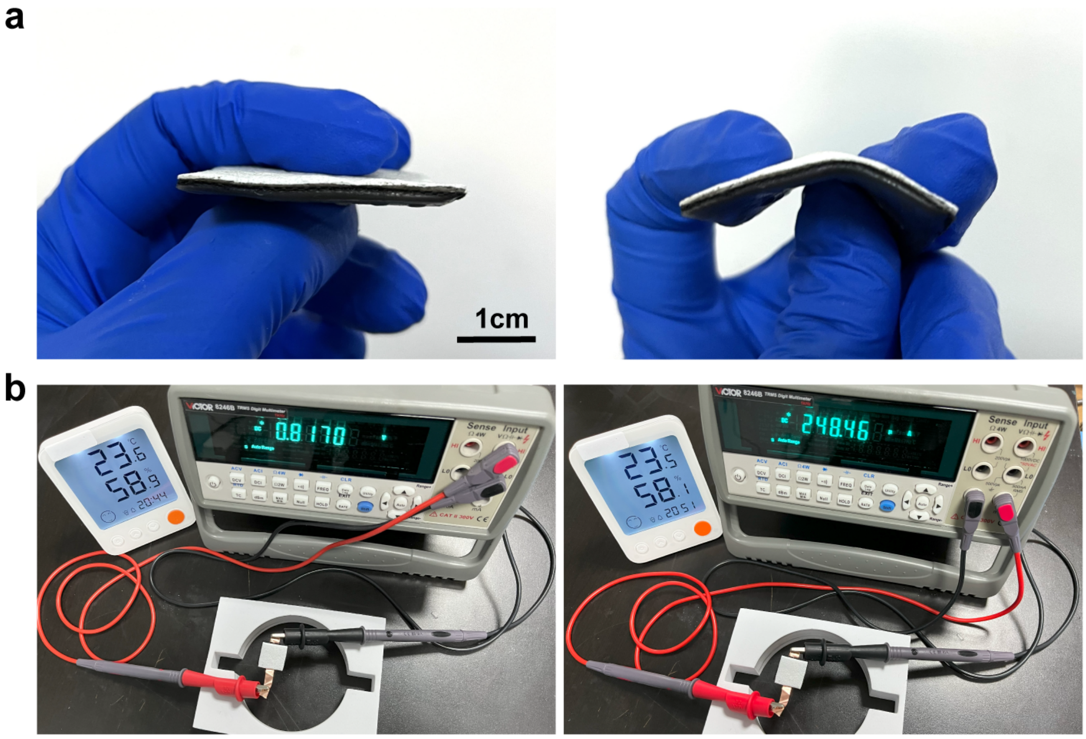

Supplementary Fig. 4 a Photographs of the PP/IH. **b** Photographs of the open-circuit voltage (left) and short-circuit current (right) output of a single PP/IH unit (1 cm^2).

Supplementary Note 2

The average solar reflectance of porous P(VdF-HFP) films with thicknesses of 1, 1.5, 2, and 2.5 mm are 85.9%, 89.5%, 95.7%, and 96.0%, respectively (Supplementary Fig. 5). Given the minimal difference in reflectance between the 2- and 2.5-mm-thick films, and the disadvantage of increased thickness for moisture desorption, a 2-mm-thick P(VdF-HFP) film was selected for subsequent experiments.

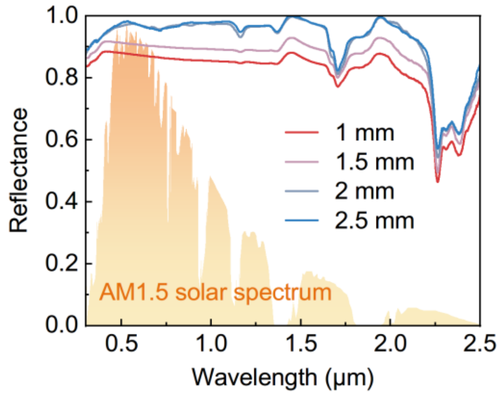

Supplementary Fig. 5 Solar reflectance spectra of porous P(VdF-HFP) films with different thicknesses.

Supplementary Note 3

To illustrate the presence of multiple absorption peaks in the atmospheric window (8–13 μm, corresponding to 770–1250 cm^{-1}) due to the various vibration modes of P(VdF-HFP) molecules, we performed Fourier transform infrared (FTIR) spectroscopy analysis. As shown in the FTIR spectrum of the porous P(VdF-HFP) film (Supplementary Fig. 6), the characteristic peaks at 1400, 1176, and 1067 cm^{-1} correspond to CH_2 wagging vibration [$\omega(CH_2)$], antisymmetric CF_2 stretching [$\nu_a(CF_2)$], and CF_3 out-of-plane deformation [$\gamma(CF_3)$], respectively. The amorphous band of P(VdF-HFP) is located at 873 cm^{-1}, while the band observed at 836 cm^{-1} belongs to the β-phase of P(VdF-HFP)[3].

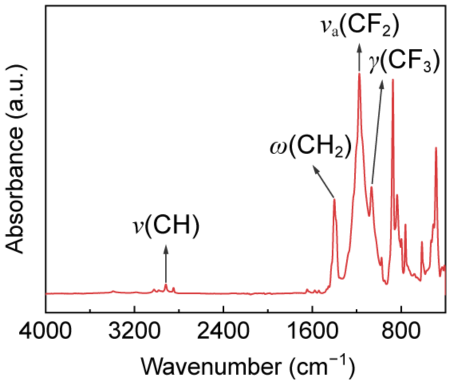

Supplementary Fig. 6 The FTIR spectrum of the hierarchically porous P(VdF-HFP) film.

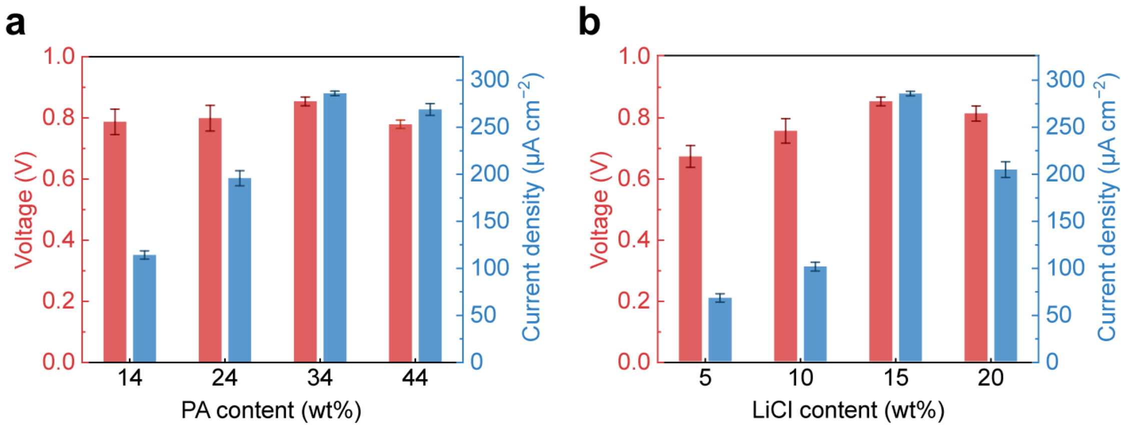

Supplementary Fig. 7 The effects of **a** PA and **b** LiCl content on the open-circuit voltage and short-circuit current, respectively. The testing conditions are 25 ℃ and 75% RH. Error bars represent the standard deviation (n = 4).

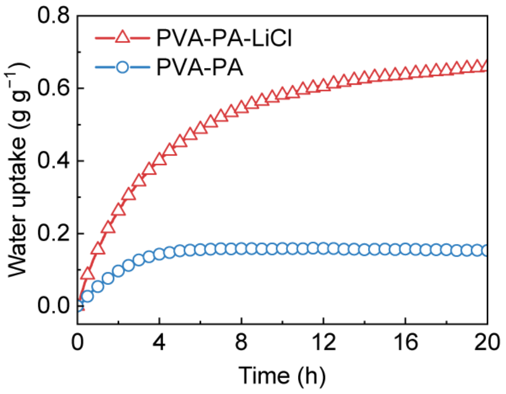

Supplementary Fig. 8 The moisture uptake capability with time after the dried hydrogels are exposed in the atmosphere (20 °C, 90% RH).

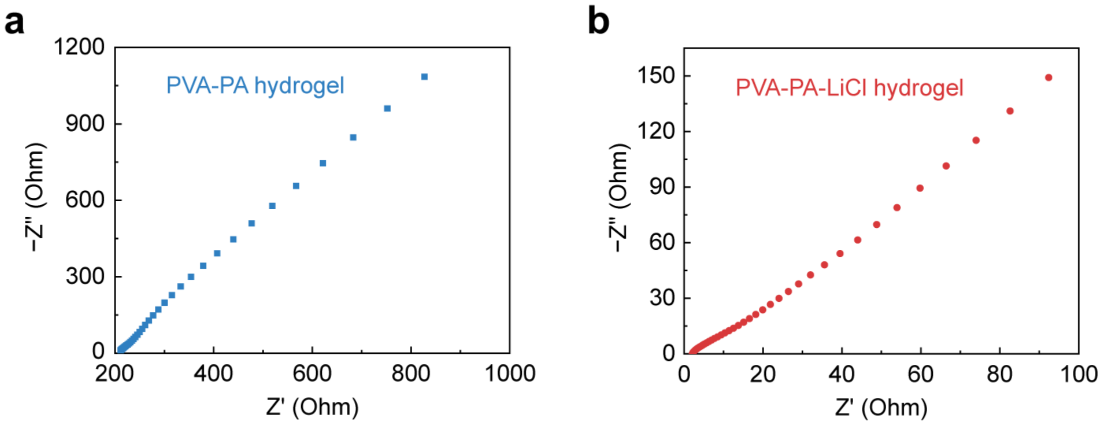

Supplementary Fig. 9 a, **b** The enlarged electrochemical impedance spectra of hydrogels with and without LiCl at 25 °C, 70% RH. The electrochemical impedance spectra were performed in a frequency range 0.1–100 kHz, with 10 mV a.c. amplitude.

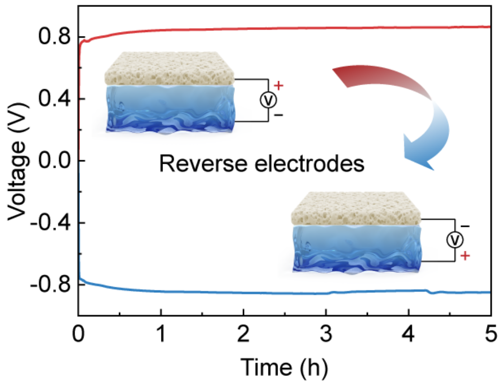

Supplementary Fig. 10 Polarity test of the PP/IH. The direction of moisture gradient in the hydrogel determines the direction of ion transport, where the lower-moisture upper side shows higher electric potential.

Supplementary Note 4

Due to the influence of thickness on the ion concentration gradient and ion diffusion distance, both V_{oc} and I_{sc} initially increase with thickness and then decrease (Supplementary Fig. 11a). The optimal output is achieved at a thickness of 2 mm. As the device size increases from 0.25 cm^2 to 4 cm^2, there is minimal change in V_{oc}, while I_{sc} gradually increases (Supplementary Fig. 11b). Enlarging the device size can be regarded as having multiple units in parallel. Therefore, adjusting the electrical output to meet various usage requirements can be flexibly achieved by controlling the device size and the series-parallel connection of device units (Fig. 5d).

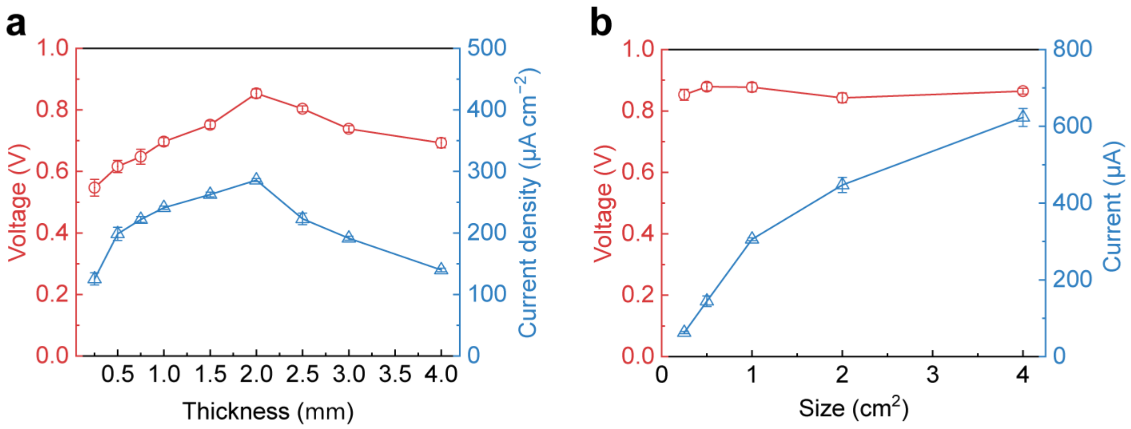

Supplementary Fig. 11 a The V_{oc} (red) and I_{sc} (blue) output of the PP/IH (1 cm^2) with different thicknesses (25 °C, 70% RH). Error bars represent the standard deviation (n = 4). **b** Electrical output plotted against the size of 2-mm-thick device (25 °C, 70% RH). Error bars represent the standard deviation (n = 4).

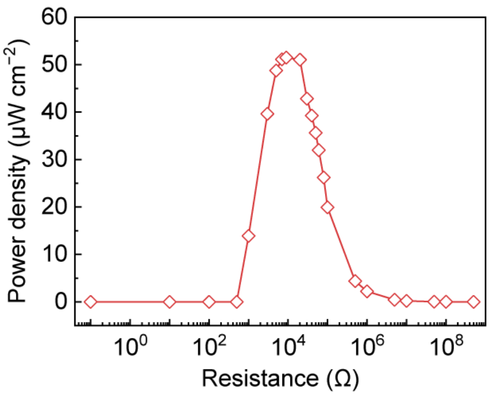

Supplementary Fig. 12 The relationship between power density and electrical resistance of the external circuit according to Fig. 3e (25 °C, 70% RH).

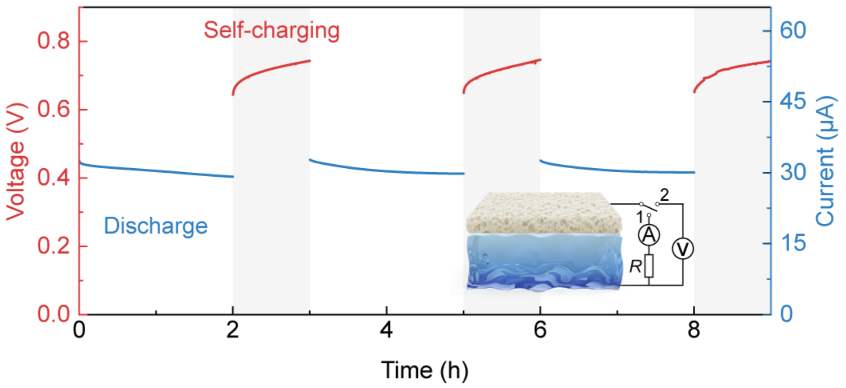

Supplementary Fig. 13 Variations of voltage and current from a working PP/IH device with a switch and an external load (R = 50 kΩ) at 25 °C and 50% RH. The inset depicts the equivalent test circuit diagram, where connections to terminals 1 and 2 correspond to discharge and self-charging processes, respectively.

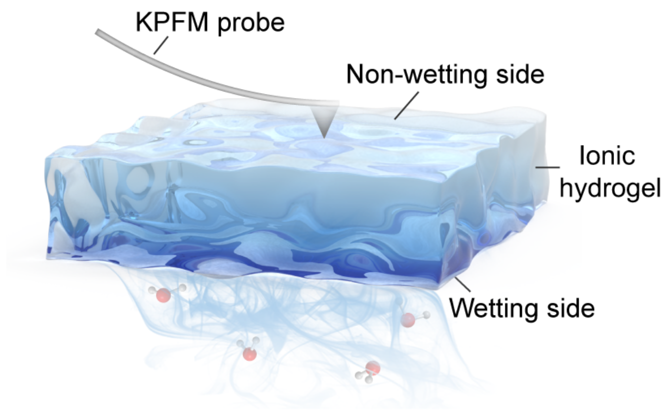

Supplementary Fig. 14 Schematic diagram of the setup for the Kelvin probe force microscope (KPFM) test.

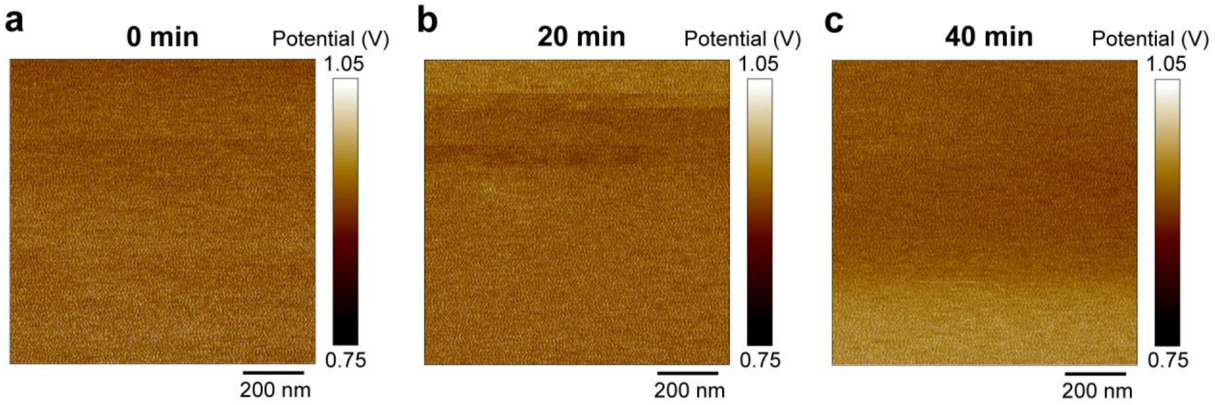

Supplementary Fig. 15 The potential acquired from KPFM of the side wetted after **a** 0 min, **b** 20 min, and **c** 40 min.

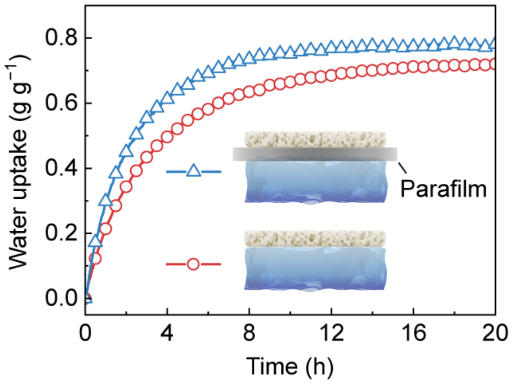

Supplementary Fig. 16 Mass change comparison of the device with the hydrophobic side sealed by a parafilm to prevent desorption (blue) and the device without any additional refitment (red) at 25 °C, 90% RH. The insets show the corresponding devices with and without refitment.

Supplementary Note 5

As depicted in Supplementary Fig. 17, the chloride distribution on the lower surface of the ionic hydrogel remained nearly constant after seven days of operation, while the lithium content on the upper surface only slightly increased. Combining the comparison of electrical output performance of hydrogels with different compositions in Fig. 2d, it can be inferred that while there is directed migration of Li^+ ions, significant improvement in current is observed only when PA and LiCl are introduced simultaneously. Therefore, the predominant migratory ions are H^+ ions dissociated from PA with six esterified phosphoric acids.

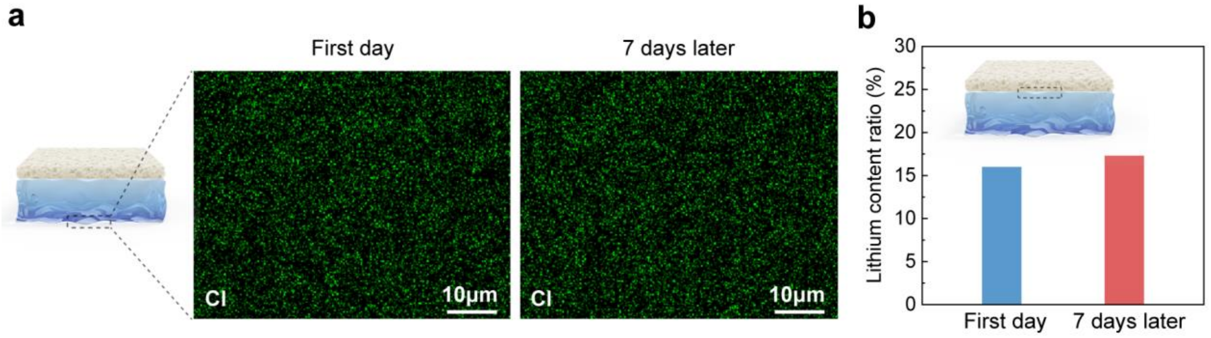

Supplementary Fig. 17 a Element mapping images of energy dispersive spectrum of chlorine on the lower surface of the ionic hydrogel and **b** the variation in the upper surface lithium content ratio after 7-day operation.

Supplementary Note 6

As shown in Supplementary Fig. 18, the prepared 2-mm-thick ionic hydrogel demonstrates an average solar absorptance of 0.41, while the average solar absorptance of blackbody-like film is 0.96. The average thermal emissivity of ionic hydrogel and blackbody-like film in the atmospheric window is 0.96 and 0.70, respectively.

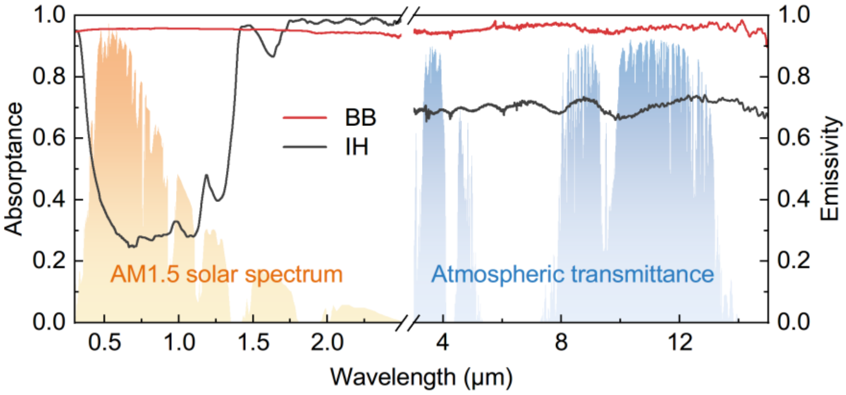

Supplementary Fig. 18 The optical properties of a 2-mm-thick ionic hydrogel (IH) and a 2-mm-thick blackbody-like film (BB).

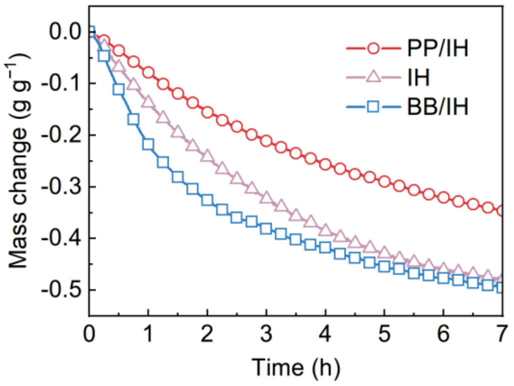

Supplementary Fig. 19 Mass change comparison of the PP/IH, BB/IH, and IH devices under simulated one sun (1 kW m^{-2}) illumination.

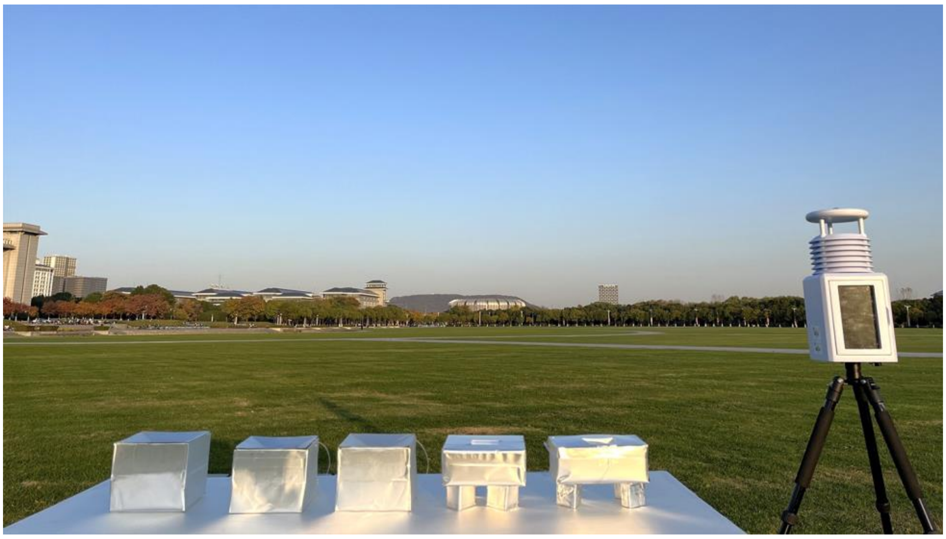

Supplementary Fig. 20 Photograph of outdoor experiment setups used to measure electrical performance and temperature of the samples. The three devices on the left were employed to measure the electrical output and temperature of PP/IH, BB/IH, and IH devices, while the two devices on the right are utilized to measure the temperature of isolated PP and BB film.

Supplementary Note 7

We employed a 1-D steady-state combined heat and mass transfer energy balance to simulate the changes in hydrogel weight and mass flux (Supplementary Fig. 21). The initial mass of the hydrogel in a device unit (1 cm^2) is 0.2323 g, with a water content of 58 wt%. This model considers factors such as solar/thermal radiation, heat conduction, water vapor diffusion, and heat and water vapor convection. The energy balance in the system can be expressed as:

$$\frac{d}{dx}(q_{\mathrm{eva}} + q_{\mathrm{rad}} + q_{\mathrm{atm}} + q_{\mathrm{sun}} + q_{\mathrm{cond+conv}}) = 0 \quad (1)$$

where q_{eva} is the evaporation energy flux, q_{rad} and q_{atm} are the emitted IR radiation and absorbed atmospheric radiation, q_{sun} and $q_{\mathrm{cond+conv}}$ are the solar absorption portion and non-radiation heat transfer flux, respectively.

The evaporative energy flux depends on the difference in the vapor mass density between the hydrogel surface ρ_c and that in the ambient ρ_{amb}. Accounting for the mass diffusion resistance in the top layer and the mass convection resistance at the air/top layer interface, the evaporation energy flux q_{eva} and the mass flux j can be calculated by:

$$q_{\mathrm{eva}} = j h_{fg} \quad (2)$$

$$j = -\frac{\rho_c - \rho_{\mathrm{amb}}}{\frac{l_{\mathrm{top}}}{D_{\mathrm{top}}} + \frac{1}{g_{\mathrm{ext}}}} \quad (3)$$

where h_{fg} is the enthalpy of vaporization of water in the hydrogel, D_{top} is the vapor diffusivity in top layer, g_{ext} is the external mass convection coefficient. The thickness of top layer l_{top} is equal to 2 mm.

When the evaporating heat absorption rate equals to the radiative cooling power with no enthalpy change for the hydrogel, the temperature of the hydrogel T_{h} can be obtained at the quasi-steady state:

$$T_{\mathrm{h}} = \frac{q_{\mathrm{rad}}(T_{\mathrm{top}})}{\frac{k_{\mathrm{top}}}{l_{\mathrm{top}}}} + T_{\mathrm{top}} \quad (4)$$

where T_{top} and T_h are the temperatures of top layer and ionic hydrogel, respectively. The thermal conductivity of top layer k_{top} obtained from the experiment is 0.062 W m^{-1} K^{-1}.

For the calculation of q_{rad}, q_{atm}, q_{sun}, and $q_{cond+conv}$, as well as further details about this model, please refer to our previous work[4].

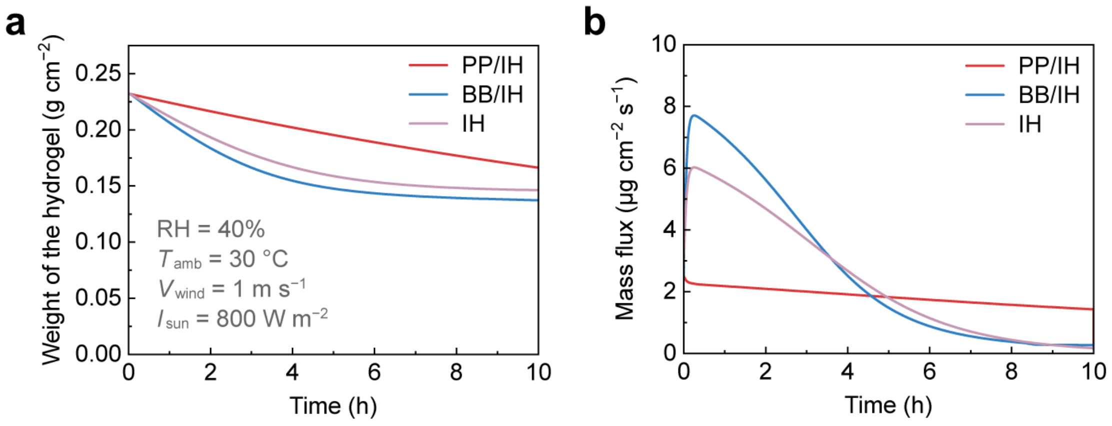

Supplementary Fig. 21 Simulated **a** variations in hydrogel weight and **b** mass flux for different devices.

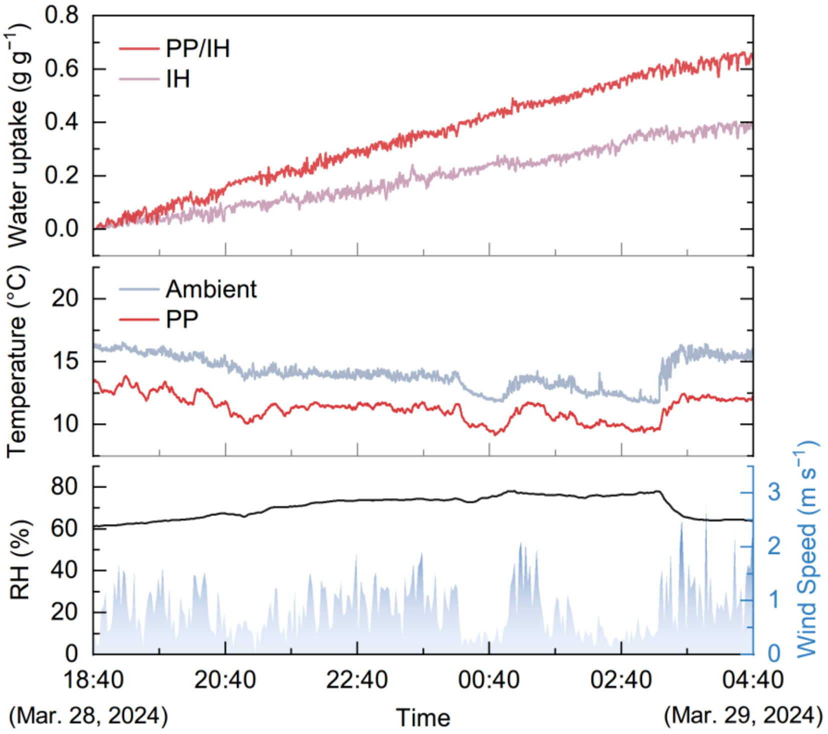

Supplementary Fig. 22 Moisture sorption curves of the PP/IH and IH devices in outdoor environment during nighttime (from 18:40 on Mar. 28 to 4:40 on Mar. 29, 2024, in Nanjing, China). Simultaneously, data on the temperature of PP film, ambient temperature, wind speed, and ambient relative humidity were recorded throughout the testing period. The hydrogels in both devices were dried before testing.

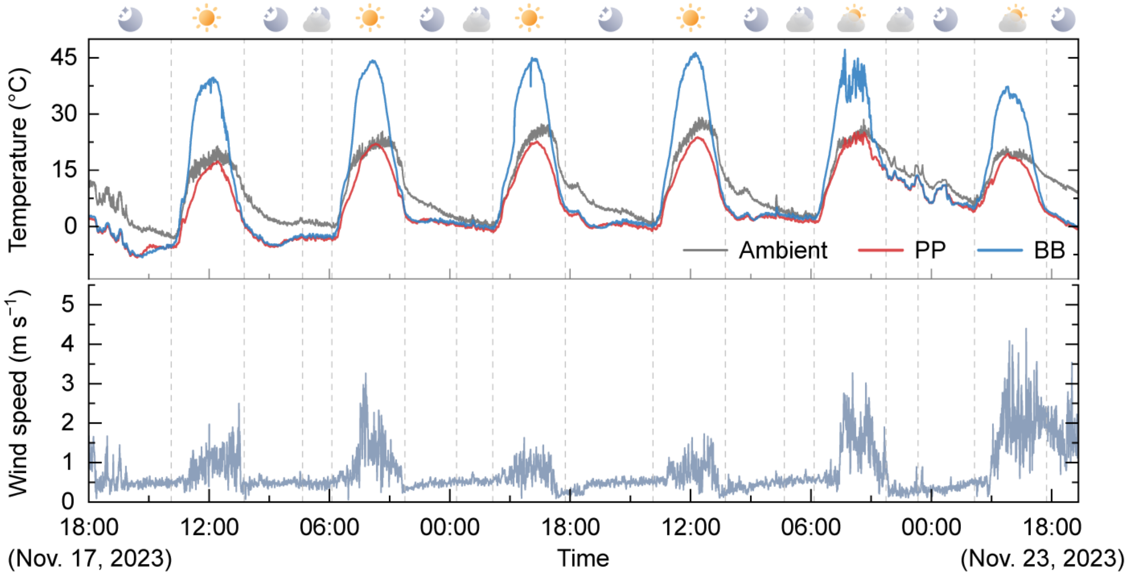

Supplementary Fig. 23 Data on temperature of isolated PP and BB film, and wind speed of the outdoor electrical performance test in Fig. 5c.

Supplementary Note 8

Attributed to the potent moisture-capturing ability of LiCl and hydrophilic functional groups, in combination with moisture storage within the porous hydrogel network, the moisture sorption capacities of the dried PP/IH over 20 hours at 40%, 60%, 80%, and 98% RH reach 0.26, 0.35, 0.58, and 0.92 g g^{-1}, respectively (Supplementary Fig. 24a). Regarding the water evaporation rate under different humidity conditions, even at a high RH of 80%, the mass change after 20 hours still exceeds 35% of that observed at 30% RH (Supplementary Fig. 24b). The test results for the moisture sorption and water evaporation behaviors of the PP/IH under different RH indicate its broad operating range and all-weather adaptability from another perspective.

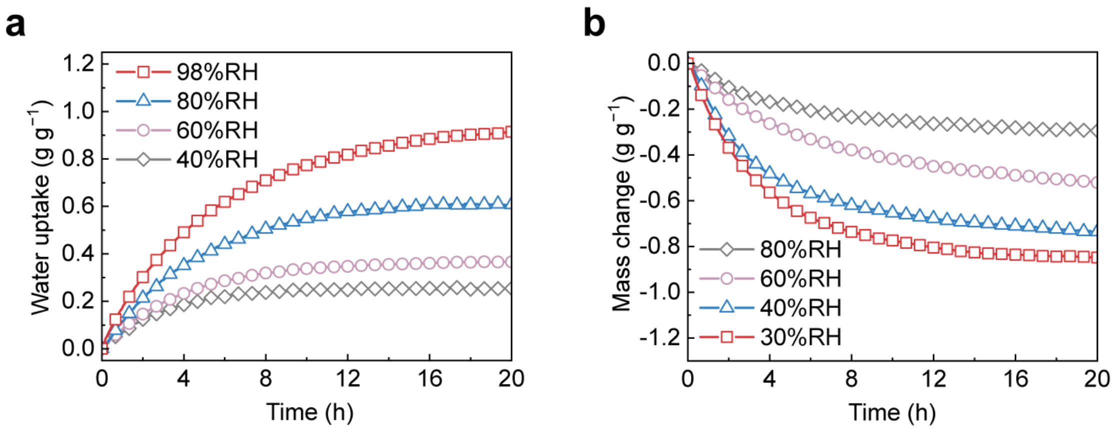

Supplementary Fig. 24 a Moisture sorption (at 20 °C) and **b** desorption (at 40 °C) curves of the PP/IH over time under different relative humidity conditions.

Supplementary Note 9

In terms of cooling efficiency, on a clear day with peak solar intensity of ~800 W m^{-2} and RH of ~40%, the PP/IH achieves an average sub-ambient cooling of ~6 ℃ and a maximum temperature drop of 8 ℃ between 10:00 and 15:00 (Supplementary Fig. 25b). In comparison, the porous P(VdF-HFP) film only exhibits a temperature reduction of 2.3 ℃. Furthermore, on a cloudy day with reduced atmospheric transparency where the radiative cooling effect is constrained, the porous P(VdF-HFP) film remains ~2.9 ℃ above the ambient temperature (Supplementary Fig. 25c). Nonetheless, the PP/IH still manages an average sub-ambient cooling of 3.4 ℃, affirming its adaptability across varying environmental conditions.

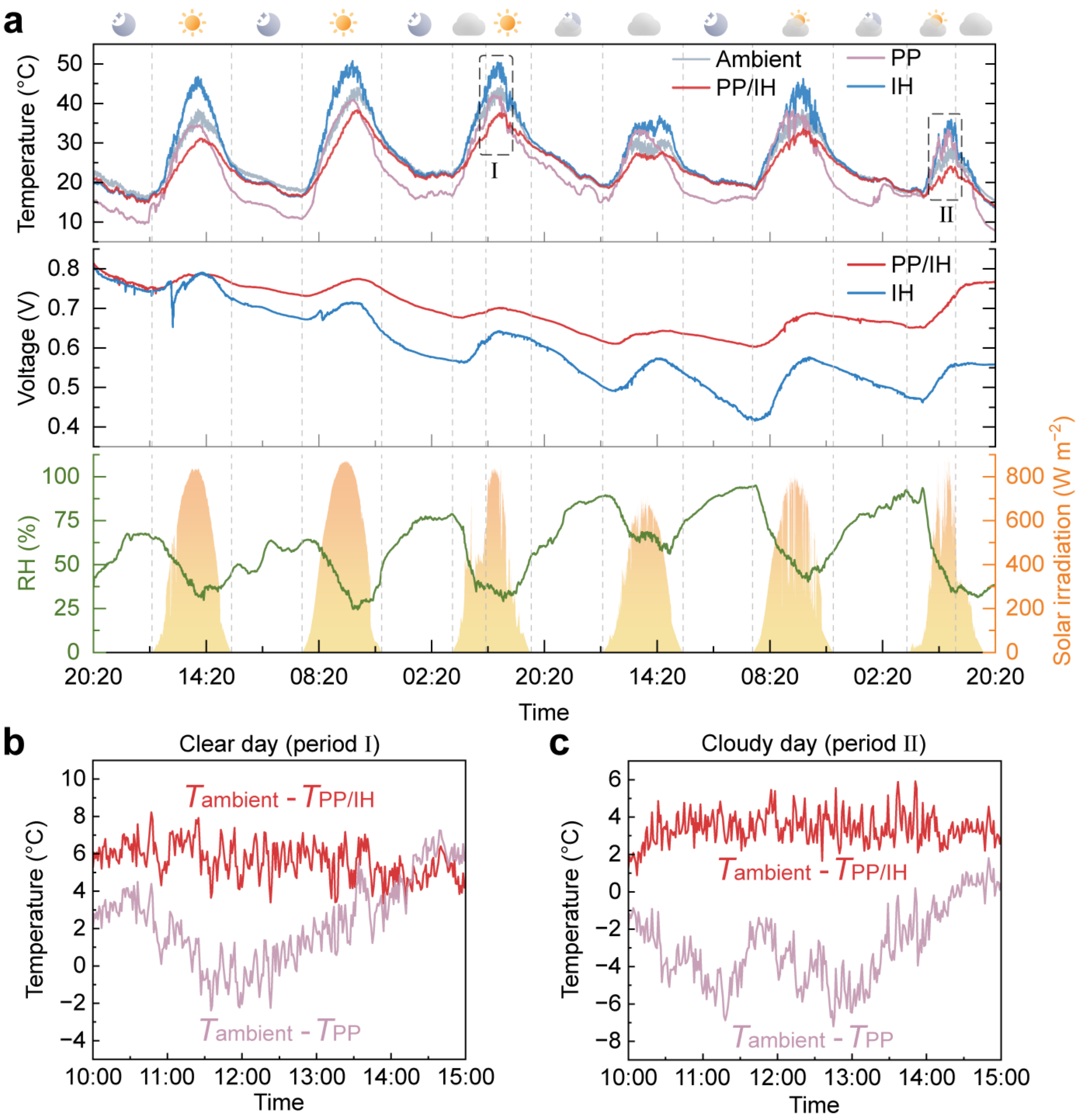

Supplementary Fig. 25 **a** Outdoor experiment conducted from 20:20 on April 15, 2023 to 20:20 on April 21, 2023, in Nanjing, China. Top panel: temperature of the PP/IH, PP, IH, and ambient; medium panel: open-circuit voltage curves of ionic hydrogel with and without the porous P(VdF-HFP) layer; bottom panel: real-time recording of solar radiation intensity and ambient relative humidity. **b** Sub-ambient temperature reduction of the PP/IH and PP samples on a clear day (period I in **a**). **c** Temperature reduction of the two samples on a cloudy day (period II in **a**).

Supplementary Table 1 The performance comparison of reported moisture-enabled generators.

Materials	Current density ($\mu A\ cm^{-2}$)	Voltage (V)	Power density ($\mu W\ cm^{-2}$)	RH (%)	Temperature (℃)	Output type	Ref.
Protein nanowire	40	0.53	5	50	room temperature	Continuous, 1500h	5
Asymmetric GO	0.9	0.45	2.02	25	20	Continuous, 500s	6
Biological nanofiber	0.022	0.1	0.0003	99	room temperature	Continuous, 167h	7
PSSA	100	0.8	0.17	80	20	Continuous, 1400s	8
GO/PAAS	12	0.6	0.07	80	25	Continuous, 120h	9
PSSA/PDDA	4	1.38	5.52	85	25	Continuous, 250h	10
PVA-PA-Glycerol	238	0.8	35	80	22	Continuous, 1018h	11
Heterogenous cellulon paper	0.83	0.78	0.7	60	25	Continuous, 10d	12
GO-grGO	0.12	1.5	—	80	room temperature	Intermittent	13
g-GOF	5	0.035	0.42	30	room temperature	Intermittent	14
TiO_2 nanowire	8	0.52	4	85	25	Intermittent	15
GO	12000	0.07	—	60	room temperature	Intermittent	16
PSS-PVA	100	0.6	7.9	85	room temperature	Intermittent	17
GO fiber	1060	0.35	—	65	room temperature	Intermittent	18
This work	306	0.88	51.5	70	25	Continuous, >7d	

Supplementary references

1 Zhao, L., Zhang, H., Tang, N., Li, M.-H. & Hu, J. Natural phytic acid-assisted polyaniline/poly(vinyl alcohol) hydrogel showing self-reinforcing features. *ACS Appl. Mater. Interfaces* **15**, 41927-41936 (2023).

2 Puziy, A. M., Poddubnaya, O. I., Socha, R. P., Gurgul, J. & Wisniewski, M. XPS and NMR studies of phosphoric acid activated carbons. *Carbon* **46**, 2113-2123 (2008).

3 Sim, L. N., Majid, S. R. & Arof, A. K. FTIR studies of PEMA/PVDF-HFP blend polymer electrolyte system incorporated with $LiCF_3SO_3$ salt. *Vib. Spectrosc.* **58**, 57-66 (2012).

4 Tang, H., Guo, C., Xu, Q. & Zhao, D. Boosting evaporative cooling performance with microporous aerogel. *Micromachines (Basel)* **14**, 219 (2023).

5 Liu, X. *et al.* Power generation from ambient humidity using protein nanowires. *Nature* **578**, 550-556 (2020).

6 Cheng, H. *et al.* Spontaneous power source in ambient air of a well-directionally reduced graphene oxide bulk. *Energy Environ. Sci.* **11**, 2839-2845 (2018).

7 Li, M. *et al.* Biological nanofibrous generator for electricity harvest from moist air flow. *Adv. Funct. Mater.* **29**, 1901798 (2019).

8 Xu, T. *et al.* An efficient polymer moist-electric generator. *Energy Environ. Sci.* **12**, 972-978 (2019).

9 Huang, Y. *et al.* All-region-applicable, continuous power supply of graphene oxide composite. *Energy Environ. Sci.* **12**, 1848-1856 (2019).

10 Wang, H. *et al.* Bilayer of polyelectrolyte films for spontaneous power generation in air up to an integrated 1,000 V output. *Nat. Nanotechnol.* **16**, 811-819 (2021).

11 Yang, S. *et al.* Ionic hydrogel for efficient and scalable moisture-electric generation. *Adv. Mater.* **34**, 2200693 (2022).

12 Tan, J. *et al.* Self-sustained electricity generator driven by the compatible integration of ambient moisture adsorption and evaporation. *Nat. Commun.* **13**, 3643 (2022).

13 Huang, Y. *et al.* Interface-mediated hygroelectric generator with an output voltage approaching 1.5 volts. *Nat. Commun.* **9**, 4166 (2018).

14 Zhao, F., Cheng, H., Zhang, Z., Jiang, L. & Qu, L. Direct power generation from a graphene oxide film under moisture. *Adv. Mater.* **27**, 4351-4357 (2015).

15 Shen, D. *et al.* Self-powered wearable electronics based on moisture enabled electricity generation. *Adv. Mater.* **30**, 1705925 (2018).

16 Cheng, H. *et al.* Flexible in-plane graphene oxide moisture-electric converter for touchless interactive panel. *Nano Energy* **45**, 37-43 (2018).

17 Wang, H. *et al.* Transparent, self-healing, arbitrary tailorable moist-electric film generator. *Nano Energy* **67**, 104238 (2020).

18 Liang, Y. *et al.* Self-powered wearable graphene fiber for information expression. *Nano Energy* **32**, 329-335 (2017).
