# Supplementary material for: Radiative cooling assisted self-sustaining and highly efficient moisture energy harvesting
Source: Nat Commun. 2024 Jul 19;15:6100. doi: 10.1038/s41467-024-50396-9 (PMC11271565; doi:10.1038/s41467-024-50396-9)
Supplement: Supplementary file 3 — Description of Additional Supplementary Files [file 41467_2024_50396_MOESM3_ESM.pdf]

Description of Additional Supplementary Files

File Name: Supplementary Movie 1
Description: A 2.9-inch electronic ink screen directly driven by the PP/IH units with a 3 × 6 series-parallel connection
